# Book Review: Caring for the people of the clouds: Aging and dementia in Oaxaca

**DOI:** 10.1177/14713012231153429

**Published:** 2023-01-25

**Authors:** Adele van Wyk

**Affiliations:** King’s College London, UK

While there is a growing awareness and understanding of dementia, the evidence regarding cultural understanding of dementia in the global south is lacking. In *Caring for the people of the clouds: Aging and dementia in Oaxaca*^1^, Yahalom presents a detailed account of the lived experience of caregiving for dependent elders living with dementia in Teotitlàn (a rural ‘indigenous’ community 20 miles from Oaxaca City, Mexico), with attention to how this experience is constituted within, and in response to, the changing surrounding social world. He explores the social relations that originate in the context of caring for elders with dementia, providing insight into how these relations embody the negotiation of broader social dynamics on a daily level. Yahalom draws on existing literature about (a) what it means to care in the context of social change (Cohen, 1998; Ticktin, 2011), (b) the nature of caregiving in localities marked by absences due to migration (Parrenas, 2005; Yarris, 2017), and (c) the economies of care (Abrego, 2014; Baldassar, & Merla, 2014; Parrenas, 2015).

This work is written from a dual psychological-anthropological standpoint. Employing a theory (social constructionism) and method (ethnography) from the field of Anthropology, Yahalom demonstrates how caring for elders with forgetfulness is socially constructed and influenced by both the traditional and the modern (p. 11). In Oaxaca, Alzheimer’s disease was understood as a modern condition that occurred in response to the stresses associated with non-traditional ways of living (chapter 2).

The contents of this book are presented in an organised manner which makes it easy to follow the development of the narrative. In chapters 1-5, Yahalom explores the social construction of caregiving for elders living with dementia by eloquently narrating what he, along with his research assistant (Alex) observed in this ethnographic study and making connections with existing literature. *Chapter 1* covers three areas namely (a) social issues concerning ageing, dementia and caregiving; (b) the problem of ageing in Teotitlàn; and (c) local representations of dementia or age-related forgetfulness. It is followed by a chapter focusing on idioms of distress related to progressive dementia. *Chapter 2* discusses what behavioural symptoms caregivers notice in dementia, followed by a description of caregiver’s etiological understanding of those symptoms. This chapter further shows that carers subscribe to medical pluralism (traditional and modern approaches to care and the tension that results from such approaches). In *chapter 3*, the reader can develop an understanding of how caregivers make choices about who to (not) consult for professional help (curanderas or allopathic doctors) and the implicit power dynamics at play. *Chapter 4* focuses on caregivers’ relationships with forgetful elders. It describes the common challenges that caregivers are dealing with and how these are perceived in the context of broader social change. The author then goes on to explain how caregiving leads to a new form of relating to elders in which former roles are reversed and end the chapter by sharing some strategies that caregivers use. In *chapter 5*, Yahalom analyses how caregivers relates to their broader community. It highlights how caregivers are often forgotten, misunderstood and subject to gossip by the larger community, despite efforts to promote family cohesion as well as engage with the broader community. The final chapter draws together this work and illustrates the larger social ambivalence about ageing in Oaxaca.

*Caring for the people of the clouds* make for an easy read, accessible to family members of people with dementia, practitioners, members of the public and some individuals with dementia who continue to read. With the background provided in the preface and introduction, most readers would not require previous knowledge to benefit from this book.

The findings Yahalom described chime with the well-established body of evidence about the experience of caregivers of people with dementia even though these are largely from high income countries. With this work, he extends considerably the current understanding of the social construction of caring in other countries, highlighting the tension between traditional values and modernising societies, and how caregivers in Oaxaca are caught between honouring traditions and a modernising society.

The background that is offered at the beginning works well to describe the research setting as well as some socio-demographic details which aids the reader in the subsequent chapters. The use of photographs brings the text to life and enhances the richness of the description. Although black and white illustrations and photos adequately represent what the author want to depict, the use of colour in some instances (e.g., p. 13, p. 15, p. 27, p. 45, p. 143, p. 157) could be considered for future versions of the book, as it will showcase the use of bright colours (like the one on the cover of this book) that is characteristic of the Mexican culture. A map of Mexico and Oaxaca would also help the reader form an idea of the geographical setting of this work.

While this book provides a thoughtful contextualisation of caring for people with dementia in Oaxaca which many readers may find interesting, researchers, clinicians and social care practitioners working in Hispanic or other collectivist client groups may find this book useful.

In this work, Yahalom has skilfully brought together clinical experience, real-life scenarios and existing literature. He should be commended for this work that has significantly broadened my understanding of the profound ways that culture dynamically constitutes human experience.

## ORCID iD

Adele van Wyk https://orcid.org/0000-0002-3310-1604

## Note

1. “People of the clouds” references a former practice of worshipping royal ancestors who, after passing, were believed to transform into divine spirits - clouds - that resided in the sky to protect the community (see Marcus, & Flannery, 1996)
